# Neutron and X-ray Diffraction Investigation of Crystal Structure and Phase Transition for Na_2/3_[Ni_y_MnzAl_1-y-z_]O_2_ as a Cathode Material for Sodium-ion Batteries

**DOI:** 10.1063/4.0000923

**Published:** 2025-10-27

**Authors:** Anthony T Pacileo, Patrick Deegan, Hao Liu

**Affiliations:** Binghamton University, Binghamton, NY, USA

## Abstract

Sodium-ion batteries promise to provide low cost and environmentally friendly energy storage as an alternative to lithium-ion batteries. The P2 layered structured Na_2/3_[Ni_1/3_Mn_2/3_]O2 material offers a high theoretical capacity but suffers rapid fade in capacity due to the P2 to O2 structure transition. Partial substitution of the transition metal ions with aluminum ions has been demonstrated to improve its cycling stability, yet, it is unclear how different substitution strategies (aliovalent vs. isovalent substitution) affects the crystal structure and the phase evolution of this material. Using joint X-ray and neutron powder diffraction methods, we have elucidated how different aluminum substitution strategies affect the Na/vacancy and Ni/Mn orderings. The aliovalent Al substitution disrupts the Na/vacancy ordering but not the transition metal ion ordering. Operando XRD revealed a reduced structural mismatch during the phase transition as a result of Al substitution.

